# Non-Genomic Estrogen Regulation of Ion Transport and Airway Surface Liquid Dynamics in Cystic Fibrosis Bronchial Epithelium

**DOI:** 10.1371/journal.pone.0078593

**Published:** 2013-11-04

**Authors:** Vinciane Saint-Criq, Sung Hoon Kim, John A. Katzenellenbogen, Brian J. Harvey

**Affiliations:** 1 Molecular Medicine Department, Royal College of Surgeons in Ireland, Beaumont Hospital, Dublin, Ireland; 2 Department of Chemistry, University of Illinois at Urbana-Champaign, Urbana, Illinois, United States of America; II Università di Napoli, Italy

## Abstract

Male cystic fibrosis (CF) patients survive longer than females and lung exacerbations in CF females vary during the estrous cycle. Estrogen has been reported to reduce the height of the airway surface liquid (ASL) in female CF bronchial epithelium. Here we investigated the effect of 17β-estradiol on the airway surface liquid height and ion transport in normal (NuLi-1) and CF (CuFi-1) bronchial epithelial monolayers. Live cell imaging using confocal microscopy revealed that airway surface liquid height was significantly higher in the non-CF cells compared to the CF cells. 17β-estradiol (0.1–10 nM) reduced the airway surface liquid height in non-CF and CF cells after 30 min treatment. Treatment with the nuclear-impeded Estrogen Dendrimer Conjugate mimicked the effect of free estrogen by reducing significantly the airway surface liquid height in CF and non-CF cells. Inhibition of chloride transport or basolateral potassium recycling decreased the airway surface liquid height and 17β-estradiol had no additive effect in the presence of these ion transporter inhibitors. 17β-estradiol decreased bumetanide-sensitive transepithelial short-circuit current in non-CF cells and prevented the forskolin-induced increase in ASL height. 17β-estradiol stimulated an amiloride-sensitive transepithelial current and increased ouabain-sensitive basolateral short-circuit current in CF cells. 17β-estradiol increased PKCδ activity in CF and non-CF cells. These results demonstrate that estrogen dehydrates CF and non-CF ASL, and these responses to 17β-estradiol are non-genomic rather than involving the classical nuclear estrogen receptor pathway. 17β-estradiol acts on the airway surface liquid by inhibiting cAMP-mediated chloride secretion in non-CF cells and increasing sodium absorption via the stimulation of PKCδ, ENaC and the Na^+^/K^+^ATPase in CF cells.

## Introduction

17β-estradiol (E2) is the most potent circulating estrogen hormone during the reproductive years of women. Beyond its role in reproduction and during the menstrual cycle, it has been shown to modulate many physiological processes such as cell proliferation, apoptosis, inflammation and ion transport in many tissues. Estrogen targets ion transport in different ways, including the control of channels mRNA and protein expression, surface abundance, degradation and activity (conductance).

In the kidney, estrogen regulates protein expression of the Na^+^-K^+^-2Cl^−^ cotransporter (NKCC2) that regulates sodium transport along the thick ascending limb of Henle's loop [1]. E2 inhibits Na^+^-Pi cotransport across renal brush border membranes from ovariectomized rats [2], induces a rise in cytosolic Ca^2+^ concentration [3] and regulates the Mg^2+^ channel TRPM6 [4], [5] and Ca^2+^ channel TRPV5 [6]. Estrogen also modulates intestinal epithelial Cl^−^ transport [7], [8], [9]. In distal colonic cells, physiological concentrations of estrogen increase intracellular Ca^2+^ concentration [10], [11] and activate c-AMP dependent protein kinase (PKA) and protein kinase C (PKC) [12]. These kinases associate with and inhibit the potassium channel KCNQ1 and [13]. Potassium recycling via KCNQ1 channels at the basolateral plasma membrane provides the driving force for chloride secretion at the apical plasma membrane. Therefore, in distal colonic cells estrogen inhibition of KCNQ1 activity leads to the inhibition of transepithelial chloride secretion. Furthermore, in the T84 epithelial cell line, inhibition of forskolin induced Cl^−^ secretion by supra-physiological concentrations of E2 appeared to result from a direct interaction with the Cl^−^ channel Cystic Fibrosis Transmembrane conductance Regulator (CFTR) protein [14].

The role of E2 in lung physiology and pathophysiology is poorly understood although in the airways, female gender enhances the risk of worse prognosis in pulmonary diseases such as asthma, chronic obstructive pulmonary disease or cystic fibrosis [15].

Cystic Fibrosis (CF) is the most frequent recessive disease in Caucasian populations and is caused by mutations in the CFTR gene leading to the absence or the lack of function of this ABC transporter-class ion channel, which transports chloride and bicarbonate. This disease affects many organs that produce exocrine secretions, but the foremost cause of morbidity and mortality is the lung pathology. In normal airways, the epithelium is covered by an aqueous film termed the Airway Surface Liquid (ASL) composed by the periciliary layer in which cilia beat to remove inhaled particles and pathogens trapped in the mucus layer. In CF, the ASL layer height is reduced below 7 µm, which compromises the efficiency of mucociliary clearance. This height of 7 µm is critical for an efficient mucociliary clearance as it is the approximate height of outstretched cilia and it has been shown that a height below 7 µm reduces mucociliary clearance [16]. Significant differences have been reported in the progression of CF in male and female patients and are termed “the CF gender gap”. Lung function among female patients deteriorates 26% more rapidly than in male patients, and on average male patients survive 9 years longer than females [17], [18]. This points to an endocrine involvement in the regulation of the processes that impact on CF severity.

Recently, Coakley *et al.* showed that E2 reduced significantly ASL height and that UTP-mediated Cl^−^ secretion is decreased in bronchial epithelium from CF and non-CF women during the period of high blood estrogen levels. This effect involves the inhibition of Ca^2+^ signalling, possibly through the binding of E2 to the Estrogen Receptor ERα but not ERβ [19]. In isolated alveolar epithelial cells from rat foetuses, E2 and progesterone increased ENaC and Na^+^/K^+^ATPase activities after 48 to 72 hours of treatment. This effect involved the stimulated expression of α- and β-ENaC and Na^+^/K^+^ATPase-β1 mRNA [20].

Besides modulating ion transport in the airways, E2 has been shown to influence the immune response in CF airways. Estrogen exerts differential effects on inflammation processes. In CF female bronchial epithelium, E2 decreases IL-8 secretion through ERβ modulation of secretory leucoprotease inhibitor and NF-κB signalling pathways, providing a propitious environment for infection and colonization [21]. In CF, female gender is also a risk factor for the development of mucoid *Pseudomonas aeruginosa* [22]. Studies on wild type and CFTR^−/−^ mice showed that female mice displayed much higher susceptibility to *P. aeruginosa* lung infection than males, (i.e., higher weight loss, number of bacteria remaining in the lungs, mortality) [23]. Moreover, CF male mice treated with estrogen showed an aggravation of *P. aeruginosa*, induced pneumonia, an up-regulation of the immune adaptative Th17 response and a decrease in the innate antibacterial immune response compared to untreated CF male mice [24].

From these studies it appears that estrogen is deleterious to CF female lung function and that the deterioration of the CF condition seen in girls at puberty is due to an elevation of estrogen levels in the body. However, the molecular mechanisms and ion transporter targets for estrogen in airway epithelium are unknown. In this study we uncover an anti-secretory and pro-absortive effect of estrogen in non-CF and CF airway epithelia, respectively. These responses occur via inhibition of Cl^−^ transport and K^+^ recycling (anti-secretion in non-CF) and stimulation of ENaC and Na^+^/K^+^ ATPase (in CF) that decrease ASL hydration in both cell types. These ion transporter and ASL responses to estrogen occur as a non-genomic effect via ERα and may underlie estrogen effects *in vivo* to exacerbate lung function especially in CF, when secretion is already compromised through the lack of functional CFTR.

## Materials and Methods

### Ethics statement

Ethical approval, for the use of primary tissue obtained from bronchoscopy, was granted by Our Lady's Children Hospital Crumlin research ethics committee and full written informed consent was obtained from the parents of all participating subjects. CF children were excluded from this study if they were taking leukotriene receptor antagonists or treatment dose with antibiotics in the two weeks prior to bronchoscopy. Primary cells were obtained from bronchial brushings obtained in 3 children (female, age range: 4 to 6 years old) with CF (all were heterozygous for the F508del mutation, recruited under the SHIELD (Study of Host Immunity and Early Lung Disease in Children with Cystic Fibrosis) Study directed by Dr Paul McNally, National Children's Hospital Crumlin, Dublin.

### Chemicals

Texas-Red dextran and Calcein AM were purchased from Biosciences (Biosciences, Invitrogen, molecular probes, Dun Laoghaire, Ireland). The perfluorocarbon (FC-72), used to prevent ASL evaporation, was a kind gift from 3M (3M, St Paul, USA). 17β-estradiol (cell culture tested) was purchased from Sigma (Sigma-Aldrich, Dublin, Ireland). The Chromanol HMR-1556 [(3R,4S)-(+)-N-[3-hydroxy-2,2-dimethyl-6-(4,4,4-trifluorobutoxy)chroman-4-yl]-N-methylmethanesulfonamide] is a chromanol derivate [25] with an increased potency to block KvLQT1/minK potassium channels, which was kindly provided by Dr. Uwe Gerlach (Aventis Pharma Deutschland, Frankfurt-am-Main, Germany). Rottlerin was purchased from Calbiochem (Nottingham, UK). The Estrogen Dendrimer Conjugate and its control molecule, the Empty dendrimer were synthesized as previously described [26], [27], stored in methanol at –20°C and used within 3 months of preparation.

### Polarized cell culture

A normal cell line called NuLi-1 (Normal Lung, University of Iowa), which was derived normal human airway epithelium of normal genotype, and a CF cell line, called CuFi-1 (CysticFibrosis, University of Iowa), which was derived from bronchial epithelium of a homozygous CFTR F508del/F508del individual, were generous gifts of A. Klingelhutz, P. Karp, and J. Zabner (University of Iowa, Iowa City, IA) [28]. Cells were initially grown to confluency in human placental collagen type VI (Sigma-Aldrich)-coated flasks using Bronchial Epithelium Basal Medium (BEBM, Clonetics™, Lonza, Isis, Bray, Ireland) supplemented with 0.1% human recombinant epidermal growth factor, 0.4% bovine pituitary extract, 0.1% epinephrine, 0.1% transferrin, 0.1% insulin, 0.1% retinoic acid, 0.1% hydrocortisone, 0.1% triiodothyronine, and 50 µg/ml gentamicin (Gibco®, Invitrogen, Dun Laoghaire, Ireland), 0.025 µg/ml penicillin/streptomycin and 1.25 µg/ml Fungizone® Antimycotic (Gibco®). These cell lines were then grown in a liquid/liquid interface on human placental collagen type VI millicell hanging cell culture inserts (Millipore, Billerica, USA) or on Costar Snapwell culture inserts (Corning, Dublin, Ireland) in BEGM, as previously described [28]. Once confluence was reached, cells were cultured in a 1∶1 ratio of Dulbecco’s Modified Eagle’s Medium (DMEM) and Ham’s F-12 Medium (Sigma-Aldrich) supplemented with 2 % UltroserG (Biosciences, Dun Laoghaire, Ireland), 50 units-50 µg/ml penicillin/streptomycin, 50 µg/ml gentamycin and 1.25 µg/ml amphotericin B. After 2 days in this DMEM:Ham’s F12 media, the cells were cultured at an air/liquid interface (ALI). Transepithelial electrical resistance (TEER) measurements were taken every 4–5 days using the Epithelial Volt-Ohm Meter (EVOM, World Precision Instruments, Aston, UK) according to manufacturer’s instructions and converted to 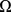.cm^−2^ based on surface area of cell culture insert. Once TEER reached at least 1000 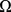.cm^−2^ (indicating the formation of a well differentiated epithelium - approximately 28 days), cells were used for experiments.

Primary cells were obtained from bronchial brushings obtained in 3 children (female, age range: 4 to 6 years old) with CF (all were heterozygous for the F508del mutation). After collection, cells were washed and seeded in flasks in tobramycin (80 µg/ml, Calbiochem, Nottingham, UK) containing BEGM. When cells reached 70% confluence, they were split and seeded onto semi-permeable support in a mixture (50:50) of tobramycin containing BEGM and retinoic acid (1 µM, Sigma-Aldrich) containing DMEM. After approximately 28 days, cells formed a ciliated well-differentiated polarized epithelium and were used in confocal microscopy experiments to measure the ASL height.

### Airway Surface Liquid height measurement

To allow the measurement of a red stained, stabilized ASL, the well-differentiated epithelium was covered with 8 µl of Texas-Red coupled to dextran (2 mg/ml; Invitrogen, Dun Laoghaire, Ireland) 24h before the experiment, as previously described [29]. The cells were stained with 5 µg/ml Calcein-AM (Molecular Probes, Bio-Sciences, Dun Laoghaire, Ireland) for 2h before the experiment, and in order to prevent ASL evaporation during the experiment, the apical surface was covered with 500 µl of perfluorocarbon (FC-72). The ASL height was measured by laser scanning confocal microscopy using a Zeiss LSM 510 (Carl Zeiss MicroImaging GmbH, Germany). Calcein green has an excitation wavelength of 488 nm and Texas-red, of 543 nm. The ASL depth was measured by taking the average XZ height of the Texas Red-stained layer at nine points in at least five different fields over the surface of the monolayer.

### Electrophysiological Measurements

NuLi-1 and CuFi-1 cell monolayers were mounted in Ussing chambers (aperture 1.12 cm^2^) and bathed in oxygenated (95% O_2_/5% CO_2_) Krebs solution [comprised of (mM): 140 Na^+^, 5.2 K^+^, 1.2 Ca^2+^, 0.8 Mg^2+^, 120 Cl^−^, 25 HCO_3_^−^, 2.4 H_2_PO4^−^, 0.4 HPO_4_^2−^, and 10 glucose)] at 37°C. Monolayers were voltage-clamped to 0 mV and monitored for changes in short-circuit current (ΔIsc) using a VCC MC8 voltage-clamp (Physiological Instruments, San Diego, CA, USA). The transepithelial short-circuit current (Isc) was recorded using Ag-AgCl electrodes in 3 M KCl agar bridges, as previously described, and the A&A software (Warner Instruments LLC, Hamden). The cells were allowed to equilibrate for 15–20 minutes before the experiments were performed. Results were normalized to an area of 1 cm^2^ and expressed as Isc ( µAmp.cm^−2^).

In these conditions, NuLi-1 and CuFi-1 cell monolayers were first treated basolaterally with E2 for 30 minutes. Then they were treated apically with amiloride, allowing the measurement of ENaC activity (represented as -ΔIsc_(amiloride)_). ATP and 

then forskolin were added and finally cell monolayers were incubated basolaterally with bumetanide. Under these conditions, ΔIsc_(bumetanide)_ across NuLi-1 or CuFi-1 monolayers is wholly reflective of changes in electrogenic Cl^−^ secretion.

Na^+^/K^+^-ATPase activity was measured as described by Lam *et al.* [30]. CuFi-1 monolayers were bathed bilaterally in low-Na^+^ (25 mM) Krebs solution, where NaCl was substituted with equimolar N-methyl-d-glucamine (NMDG)-Cl^−^. Apical membranes were permeabilized with amphotericin B (10 µM) and ouabain (100 µM) was added basolaterally. Under these conditions (i.e., in the absence of ionic gradients across the permeabilized monolayer), changes in Isc (-ΔIsc_(ouabain)_) are reflective of electrogenic transport through the Na^+^/K^+^-ATPase.

### Protein extraction

NuLi-1 and CuFi-1 cell monolayers grown under a thin film at the air-liquid interface, were washed twice with ice-cold PBS and lysed (lysis buffer composition: TrisHCl 50 mM, NaCl 150 mM, NP40 1 %, Glycerol 3 %, EDTA 2 mM, EGTA 2 mM). Lysates were sonicated (3×10s pulses), centrifuged (14 000 rpm, 15 min, 4°C) and pellets were discarded. Protein concentrations were measured using BioRad protein assay and the supernatants were stored at –80°C until used for Immunoblotting

### Immunoblotting

Samples were normalized for protein content, and 2X gel loading buffer (Sigma-Aldrich) was added. After heating at 95°C for 10 min, samples were separated by SDS-PAGE and transferred to nitrocellulose membranes. Membranes were blocked in 5% blocking buffer [Bovine Serum Albumin in Tris buffered saline with 0.1% Tween (TBST)] for 60 min at room temperature, followed by incubation with primary antibody (phospho-PKCα/βII, phospho-PKCζ/λ, phospho-PKCδ/θ, total PKCα/βII, total PKCζ/λ, total PKCδ/θ dilution 1/1000 in 5% BSA in 0.1% TBST – cell signalling or β-actin 1/5000, Sigma-Aldrich) overnight at 4°C. After washing (4×) in TBST, membranes were incubated with HRP-conjugated secondary antibodies (anti-rabbit 1/10000, Abcam, anti-mouse 1/10000) in 5% blocking buffer for 60 min at room temperature. After further washing (4×) in TBST, immunoreactive proteins were detected by enhanced chemiluminescence (Amersham Biosciences, Little Chalfont, UK).

### Statistical analysis

Data shown are means ± SEM, and the Student paired t-test or one-way repeated measure ANOVA were used to compare the means of different treatment groups unless otherwise indicated. Unpaired t-test and one-way ANOVA were used for the comparison of NuLi-1 *vs*. CuFi-1 cells. The difference between two means was considered to be significant when p<0.05.

## Results

### Estrogen decreases ASL height in normal and CF bronchial epithelial cell lines and in CF primary bronchial epithelial cells

**Figure 1:**
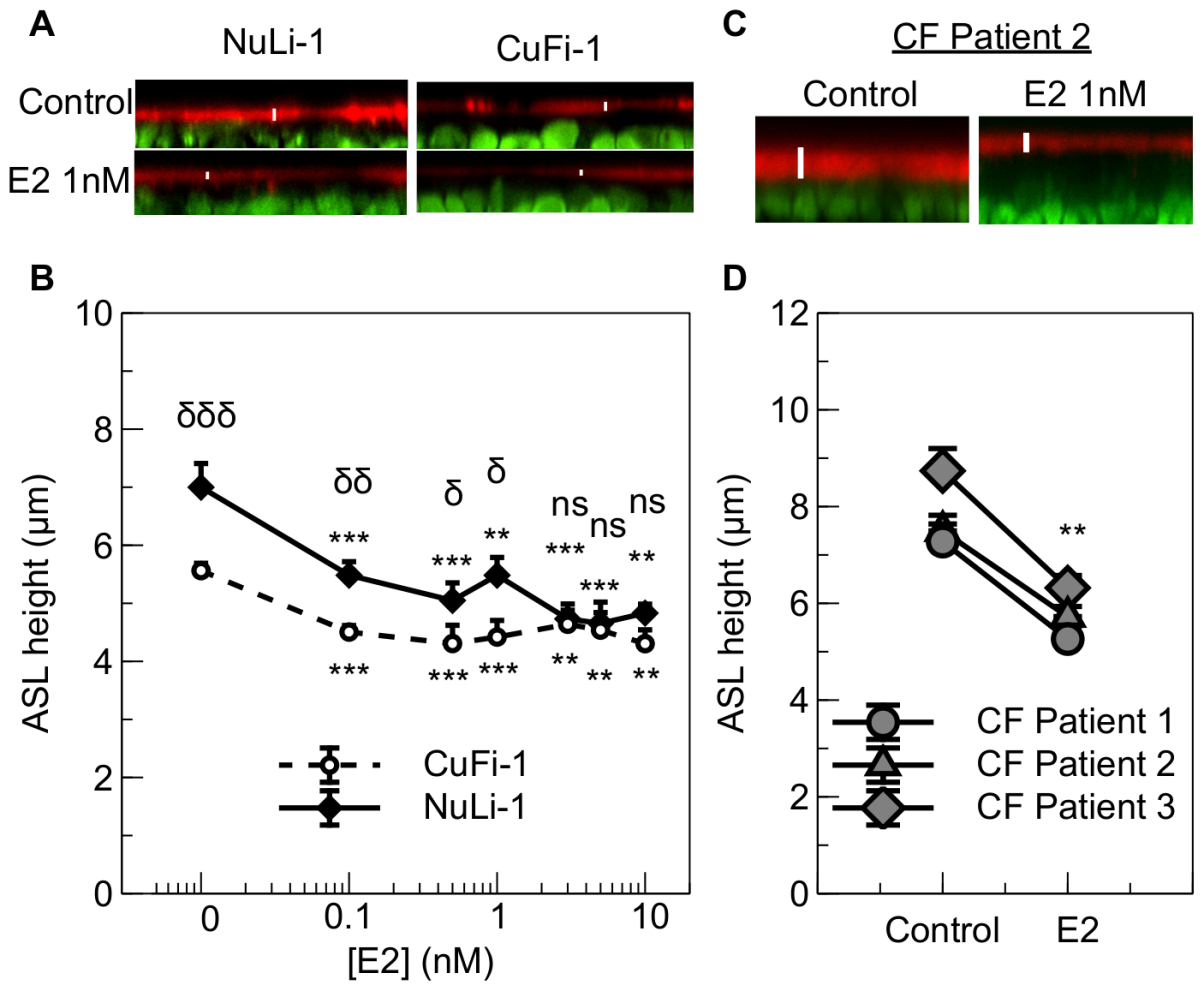
Concentration-dependence of 17β-estradiol effects on ASL height in NuLi-1 and CuFi-1 epithelial monolayers and the effect of 1 nM E2 on ASL height of primary CF bronchial epithelial cells. Epithelial cells were stained with Calcein Green and the ASL stained using dextran conjugated Texas Red™ fluorochrome 24 h before estrogen treatment. Panel A shows representative z-plane sections of NuLi-1 (left) and CuFi-1 (right) cells under basal conditions (top) and after 30 mins treatment with 1 nM E2 (bottom). Mean changes in ASL height in control conditions or following treatment with different concentrations of estrogen are shown in panel B for NuLi-1 (solid line) and CuFi-1 (dashed line) cells (n≥4, Error bars reflect standard error of the mean, ANOVA, ** p<0.01, *** p<0.001, compared to control conditions; δ p<0.05, δδ p<0.01, δδδ p<0.001, difference between NuLi-1 and CuFi-1 cells at the indicated estrogen concentration, ns: non significant). Representative z-plane sections of CF primary bronchial epithelial cells under basal conditions (left) and after 30 mins treatment with 1 nM E2 (right) (panel C) and mean changes in ASL height in primary bronchial epithelial cells of 3 CF female children (panel D) (n = 3, paired t-test, ** p<0.01)

NuLi-1 and CuFi-1 cell monolayers were treated for 30 minutes with increasing concentrations of 17β-estradiol after the ASL was stained with TexasRed™ Dextran and epithelial cells were stained with Calcein Green AM. Under basal conditions, CF epithelia showed a disrupted and thinner ASL compared to the non-CF epithelia (Figure 1A). Moreover, at a concentration as low as 0.1 nM, E2 significantly decreased ASL height in both CF and non-CF cell lines, and this effect was maximal as increasing E2 concentration did not further lower ASLh (Figure 1B). It is interesting to note that after treatment with E2, the ASLh in CuFi-1 cells is still significantly lower than in NuLi-1 cells up to a concentration of 1 nM of estradiol. These concentrations are the closest to physiological concentrations of E2. Indeed, it has been shown that the concentration of estradiol in the plasma of normal women is less than 2 nM [31]. On average, when treated with concentrations from 0.1 to 1 nM, the ASLh is 0.9±0.1 µm lower in CuFi-1 than in NuLi-1 cells, whereas it is only 0.3±0.1 µm lower when the cell monolayers are treated with concentrations from 3 to 10 nM. (n = 3, p = 0.016).

The effect of E2 on ASL height was confirmed using primary epithelial cells from bronchial brushings in 3 young CF children who were heterozygous for the F508del mutation. As shown in figure 1C, 1 nM E2 strongly decreased the ASL height in the CF airway epithelium (representative confocal image from patient 2). Figure 1D summarizes the significant decrease induced by estrogen on ASL height in the primary cultures from the 3 CF children (control: 7.8±0.5 µm, E2: 5.8±0.3 µm, n = 3, paired t-test, p = 0.008).

### Rapid and non-genomic effects of E2 on ASL height

**Figure 2:**
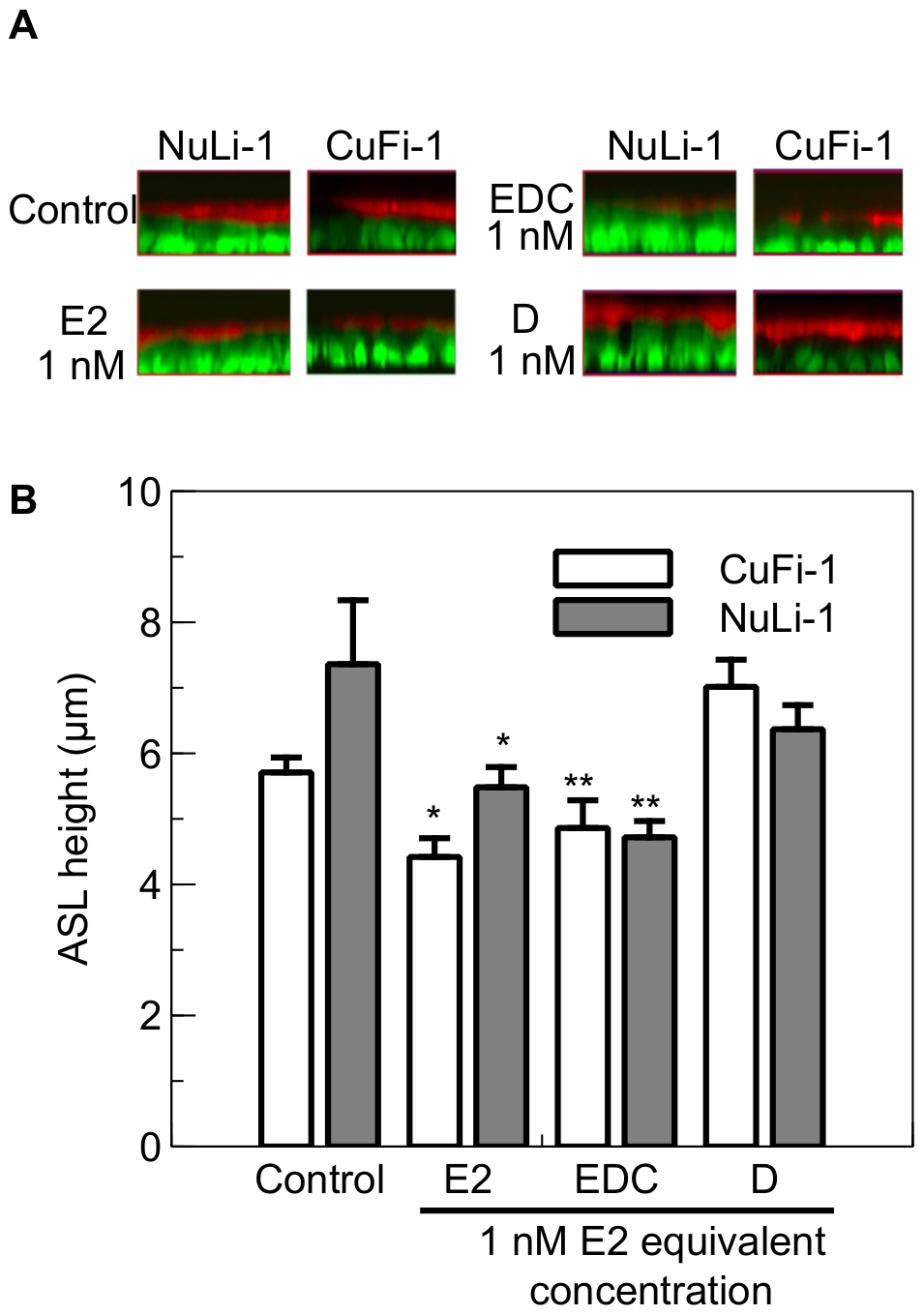
Effect of the Estrogen Dendrimer Conjugate on ASL height. NuLi-1 and CuFi-1 epithelial monolayers were treated with 1 nM E2, or EDC at concentrations providing 1 nM estrogen equivalents, or empty dendrimer at the concentration that matched that in EDC at a 1 nM estrogen equivalent. Panel A shows typical z-plane sections in control conditions or after treatment with E2, EDC or the empty dendrimer (D). The concentrations given for the EDC and the empty dendrimer are in equivalent E2 concentrations. Panel B shows the mean changes in ASL height in control conditions or following exposure to E2, EDC or the empty dendrimer (n≥4, Error bars reflect standard error of the mean, ANOVA, * p<0.05, ** p<0.01).

Non-genomic responses to steroid hormones can be discriminated from genomic actions by their rapid time course and more precisely by employing certain selective estrogen receptor modulators (SERMS) having pathway selective activity. Estrogen conjugated to cell-impenetrant macromolecules, such as Bovine Serum Albumin (E2-BSA), have largely been used for this purpose, although there have been concern about the efficiency of such molecules. Indeed, the selected binding site of the estrogen could interfere with the binding to the receptors and the weak chemical stability could lead to the release of small fragments of active molecules [32], [33]. Here, we have used a novel SERM, the Estrogen Dendrimer Conjugate (EDC), to selectively activate the extranuclear estrogen receptor. This compound is composed of a dendrimer molecule (PAMAM) that is coupled to ∼20 molecules of estrogen, allowing the EDC to interact at the plasma membrane and enter the cytoplasm but not penetrate the nuclear membrane, thus discriminating the genomic from the non-genomic effects of E2 [26], [34]. Both CF and non-CF cell lines were treated for 30 minutes either with E2, EDC or the empty dendrimer PAMAM molecule (that is not coupled to E2) as a control. After 30 minutes treatment, ASL height was significantly decreased in both cell lines by E2 (by 22.6%±5.1 in CuFi-1, p = 0.019, n = 12 and 25.5%±5.4 in NuLi-1 cells, p = 0.001, n = 6) and EDC (by 14.9%±7.4 in CuFi-1, p = 0.004, n≥5 and 35.9%±3.4 in NuLi-1 cells, p<0.001, n≥4) at 1 nM (E2 equivalent concentration), but not by the empty dendrimer (D) (Figure 2). This result indicates that E2 regulates ASL height rapidly in a non-genomic manner.

### Estrogen effects on Na^+^ absorption and Cl^−^ secretion in non-CF epithelial monolayers and consequences for ASL height

**Figure 3:**
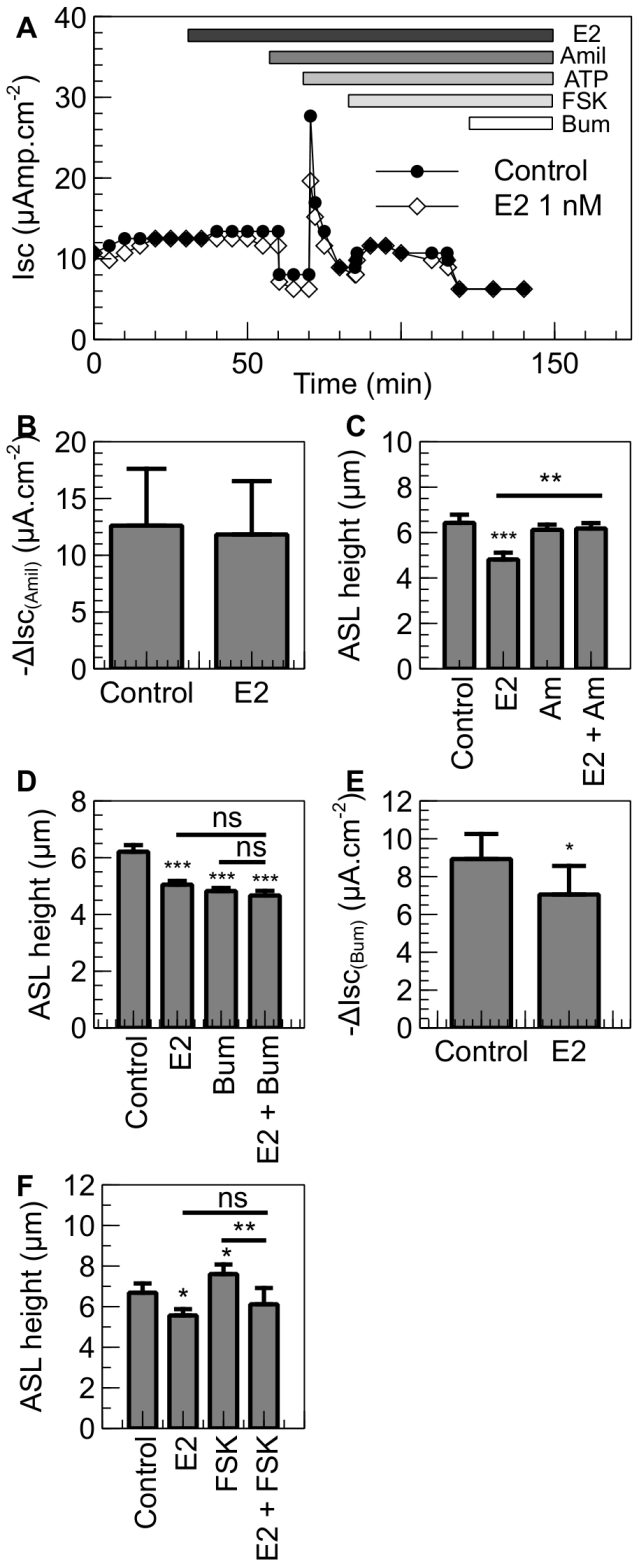
E2 inhibits Cl^−^ secretion in NuLi-1 cells. NuLi-1 cells were cultured on collagen-coated culture inserts. The monolayers were then allowed to reach a transepithelial resistance of 1,000 Ω.cm^−2^ or above prior the experiment. A. When mounted in the Ussing chambers, the cells were allowed to stabilize and were then treated basolaterally with E2. The changes in short-circuit current were then measured in response to amiloride (10 µM), ATP (100 µM), forskolin (10 µM) and bumetanide (10 µM). Typical traces of Isc in control and E2 treated NuLi-1 cells are shown. B. Summary of the amiloride short circuit current in control and E2 treated cells. Panel C shows ASLh measurement in NuLi-1 in response to estrogen and ENaC inhibition. NuLi-1 monolayers were treated with E2 (1 nM), Amiloride (Am, 300 µM) or pretreated with estrogen and then treated with amiloride (E2 + Am) (n≥3, error bars reflect standard error of the mean, ANOVA, ** p<0.01, *** p<0.001). Panels D and F show ASLh measurement in NuLi-1 epithelial cells after treatment with (D) E2 (30 min, 1 nM), Bumetanide (Bum, 30 min, 10 µM) or pretreated with E2 and then treated with Bumetanide (E2 + Bum) or (F) E2 (30 min, 1 nM), Forskolin (FSK, 30 min, 10 µM) or pretreated with E2 and then treated with Forskolin (E2 + FSK) (n≥5, Error bars reflect standard error of the mean, ANOVA, * p<0.05, ** p<0.01, *** p<0.001). Panel E shows bumetanide-sensitive current in NuLi-1 cells treated with E2 or with vehicle. Bumetanide was added after 30 minutes (n≥5, Error bars reflect standard error of the mean, Student's t test, * p<0.05).

The ability of estrogen to reduce ASL height implies an effect on transepithelial ion transport and obligatory water flow. We investigated the ion transporters potentially targeted by estrogen using transporter inhibitors and measuring their effects in combination with estrogen on ASL height and short-circuit current. In NuLi-1 cells, amiloride sensitive current is higher than in CuFi-1 cells (NuLi-1: 19.1±1.5 µAmp.cm^−2^, CuFi-1: 12.8±1.0 µAmp.cm^−2^, p<0.001, n≥17) (Figure S1A). But in these non-CF cells it represents only 70.0±3.7% of the total current whereas amiloride-sensitive current represents 86.9±2.2% of the total current in CuFi-1 cells (p<0.001, n≥17) (Figure S1B). The effect of estrogen on different ion transporters was tested in Ussing chamber experiments (Figure 3A). In short-circuit conditions, estrogen did not have any effect on amiloride-sensitive current (Control: amil Isc 12.6±5.0 µAmp.cm^−2^, E2: amil Isc 11.8±4.7 µAmp.cm^−2^, p = 0.649, n  =  4), (Figure 3B). Moreover, in open-circuit conditions, NuLi-1 cells treated with amiloride do not show an increase in ASLh (Figure 3C) (control: ASLh 6.4±0.4 µm, amiloride: ASLh 6.1±0.2 µm, p = 0.484, n = 4). This shows that even though amiloride-sensitive current represents 70% of the total current in NuLi-1 cells, the inhibition of ENaC by amiloride does not modulate ASLh in open-circuit conditions and that non-CF cells are able to finely regulate their ASLh using other pathways and ion transporters. The addition of amiloride after treatment of NuLi-1 cells with E2 shows that the inhibition of ENaC allows recovery of a similar ASLh as in control conditions (control: ASLh 6.4±0.4 µm, E2 + amiloride: ASLh 6.2±0.2 µm, p = 0.356, n  =  4) (Figure 3C). These results strongly suggest that E2 targets other ion transporters besides ENaC to regulate ASLh in non-CF cells.

Cl^−^ secretion plays a major role in generating and regulating the ASL height and transepithelial ionic current in NuLi-1 cells. In the non-CF cell line, the application of the NKCC1 inhibitor, bumetanide, to the basolateral side of NuLi-1 monolayers led to a significant decrease in ASL height (Control: ASLh 6.2±0.2 µm, Bumetanide: ASLh 4.8±0.1 µm, p<0.001, n = 5). Pretreatment of the non-CF cell monolayers with E2 had no additive effect on ASL height (E2 + Bumetanide: ASLh 4.7±0.2 µm), indicating that the hormone reduces ASL via inhibition of Cl^−^ secretion in the non-CF monolayer (Figure 3D). To confirm this result, NuLi-1 monolayers were mounted in Ussing chambers and Cl^−^ secretion was measured as the bumetanide-sensitive short-circuit current. Following treatment with E2, the bumetanide-sensitive Isc was significantly reduced by 21.0±7.7 % (p<0.05, n =  5) compared to non-treated NuLi-1 cells (Figure 3E).

Transepithelial chloride secretion was stimulated by the cAMP agonist forskolin in NuLi-1 monolayers (Figure 3A). This is a typical Isc response in CFTR-expressing secretory epithelia. We tested the effect of estrogen and forskolin on ASL height. The ASL height in NuLi-1 monolayers was significantly increased after 30 minutes forskolin treatment from 6.7±0.5 µm to 7.6±0.5 µm, n  =  4, p<0.05 (Figure 3F). Pre-treatment of NuLi-1 monolayers with estrogen (1 nM) for 30 mins prior to forskolin, prevented the stimulatory effect of this cAMP agonist on ASL height (E2 alone: ASLh 5.6±0.3 µm, FSK alone: ASLh 7.6±0.5 µm, E2 + FSK: ASLh 6.1±0.8 µm, n  =  4, p<0.01), (Figure 3F).

### Estrogen effects on Na^+^ absorption and Cl^−^ secretion in CF epithelial monolayers and consequences for ASL height

**Figure 4:**
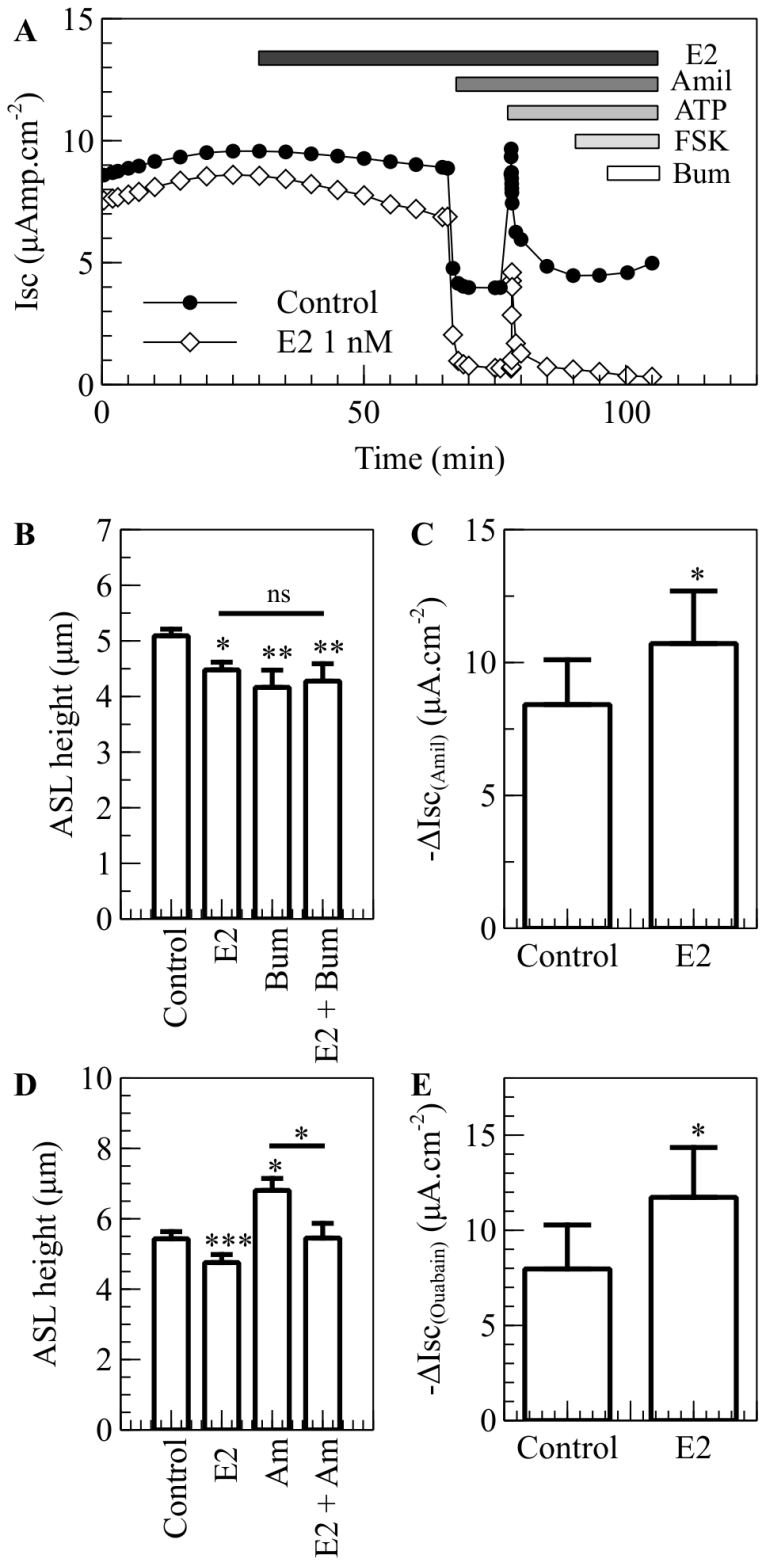
E2 increases Na^+^ absorption in CuFi-1 cells. CuFi-1 cells were cultured on collagen-coated culture inserts. The monolayers were allowed to reach a transepithelial resistance of 1,000 Ω.cm^−2^ or above prior the experiment. A. In the Ussing chambers, the cells were allowed to stabilize and were then treated basolaterally with E2. The changes in short-circuit current were measured in response to amiloride (10 µM), ATP (100 µM), forskolin (10 µM) and bumetanide (10 µM). Typical traces of Isc in vehicle and E2 treated CuFi-1 cells are shown. B. ASLh was measured in CuFi-1 cells in response to estrogen and NKCC1 inhibition. CuFi-1 monolayers were treated with E2 (1 nM), Bumetanide (Bum, 10 µM) or pretreated with estrogen before bumetanide was added (E2 + Bum) (n = 5, error bars reflect standard error of the mean, ANOVA, * p<0.05, ** p<0.01, ns: non significant). C. Amiloride-sensitive current in CuFi-1 cells treated with E2 or vehicle. Amiloride was added after 30 minutes (n = 6, Error bars reflect standard error of the mean, Student's t test, * p<0.05). D. ASLh measurement in CuFi-1 cells after treatment with E2 (30 min, 1 nM), Amiloride (Amil, 20 min, 300 µM) or pretreated with E2 and then treated with Amiloride (E2 + Amil) (n = 7, Error bars reflect standard error of the mean, ANOVA, * p<0.05, *** p<0.001). F. Ouabain–sensitive current in CuFi-1 cells mounted in Ussing chambers and bathed in modified low Na^+^ Krebs buffer in which NaCl was replaced by NMDG-Cl^−^. After the current stabilised, the apical membrane was permeabilized and the cells were treated with E2 or with vehicle and Ouabain (100 mM) was added to the basolateral chamber after 30 minutes (n = 4, Error bars reflect standard error of the mean, Student's t test, * p<0.05).

CF cells secrete very low or no Cl^−^. In short-circuit conditions, the bumetanide-sensitive current is 0.0±0.1 µAmp.cm^−2^ (Figure S1A and Figure 4A) and represents 1.0±3.0% of the total current, whereas it represents 38.4±2.5% of the forskolin-induced current in non-CF cells (p<0.001, n≥8) (Figure S1B). Although there is no bumetanide-sensitive current in CuFi-1 cells in short-circuit conditions, bumetanide alone is able to significantly decrease ASLh in open-circuit conditions (Control: ASLh 5.1±0.1 µm, bumetanide: ASLh 4.2±0.3 µm, p = 0.005, n = 5) (Figure 4B). Bumetanide is a specific inhibitor for the Na^+^-K^+^-2Cl^−^ cotransporter (NKCC1), and therefore it also inhibits Na^+^ entry in the cells. As the Na^+^/K^+^ATPase is constitutively active to maintain the membrane potential difference, the inhibition of NKCC1 might lead to an increase in Na^+^ absorption via ENaC, explaining the decrease in ASLh after bumetanide treatment in CuFi-1 cells. E2 addition prior to bumetanide treatment does not show an additive effect (ASLh: E2: 4.5±0.1 µm, E2 + Bumetanide: 4.3±0.3 µm, ns, n = 5), suggesting that E2 could target ENaC (Figure 4B).

CuFi-1 cell monolayers displayed an increased amiloride-sensitive current after treatment with E2 for 30 min (Control: Amiloride Isc 8.4±1.7 µAmp.cm^−2^, E2: Amiloride Isc 10.7±2 µAmp.cm^−2^, n  =  7, p<0.05, (Figure 4C). The consequences for ASL height regulation of estrogen stimulation of Na^+^ absorption via ENaC were tested. As reported above, 1 nM E2 significantly decreased ASL height (from Control ASLh 5.4±0.2 µm to E2 4.8±0.2 µm, n  =  6, p<0.001), whereas amiloride, diluted in the perfluorocarbon and applied on the apical surface of CuFi-1 monolayers, increased the ASL height (Control: 5.4±0.2 µm, Amiloride: 6.8±0.3 µm, n  =  6, p<0.05) (Figure 4D). These results indicate that estrogen and amiloride have opposite effects on ENaC activity, resulting in opposite effects on ASL height. This conclusion is reinforced by the observation that estrogen pretreatment of CuFi-1 monolayers prevented the amiloride-induced increase in ASL height (Amiloride: ASLh 6.8±0.3 µm, E2 + Amiloride: ASLh 5.5±0.4 µm, n  =  6, p<0.05), (Figure 4D).

### Estrogen effects on Na^+^/K^+^ ATPase in CF epithelial monolayers

Although the amiloride experiments indicate a stimulatory effect of estrogen on ENaC, the transepithelial sodium reabsorption is a two-step process, and an effect of E2 on the basolateral Na^+^/K^+^ pump may also contribute to an enhanced sodium uptake. We investigated the effect of E2 on the basolateral membrane ionic current produced by Na^+^/K^+^ ATPase activity in CuFi-1 monolayers mounted in Ussing chambers. As described in Methods, the specific current generated by the Na^+^/K^+^ ATPase pump was extracted from the total current flowing across the basolateral cell membrane by first permeabilizing the apical membrane with amphotericin B and then measuring the ouabain-sensitive short-circuit current. Exposure to estrogen caused a rapid increase (46.3%) in the ouabain-sensitive current (Control: 8.0±2.3 µAmp.cm^−2^ to E2 11.7±2.6 µAmp.cm^−2^, n = 4, p<0.05), (Figure 4E). This result implies that the stimulatory effect of estrogen on transepithelial sodium reabsorption results from a combination of enhanced ENaC and Na^+^/K^+^ ATPase activity.

### Estrogen effects on K^+^ recycling in CF and non-CF epithelial monolayers and consequences for ASL height

**Figure 5:**
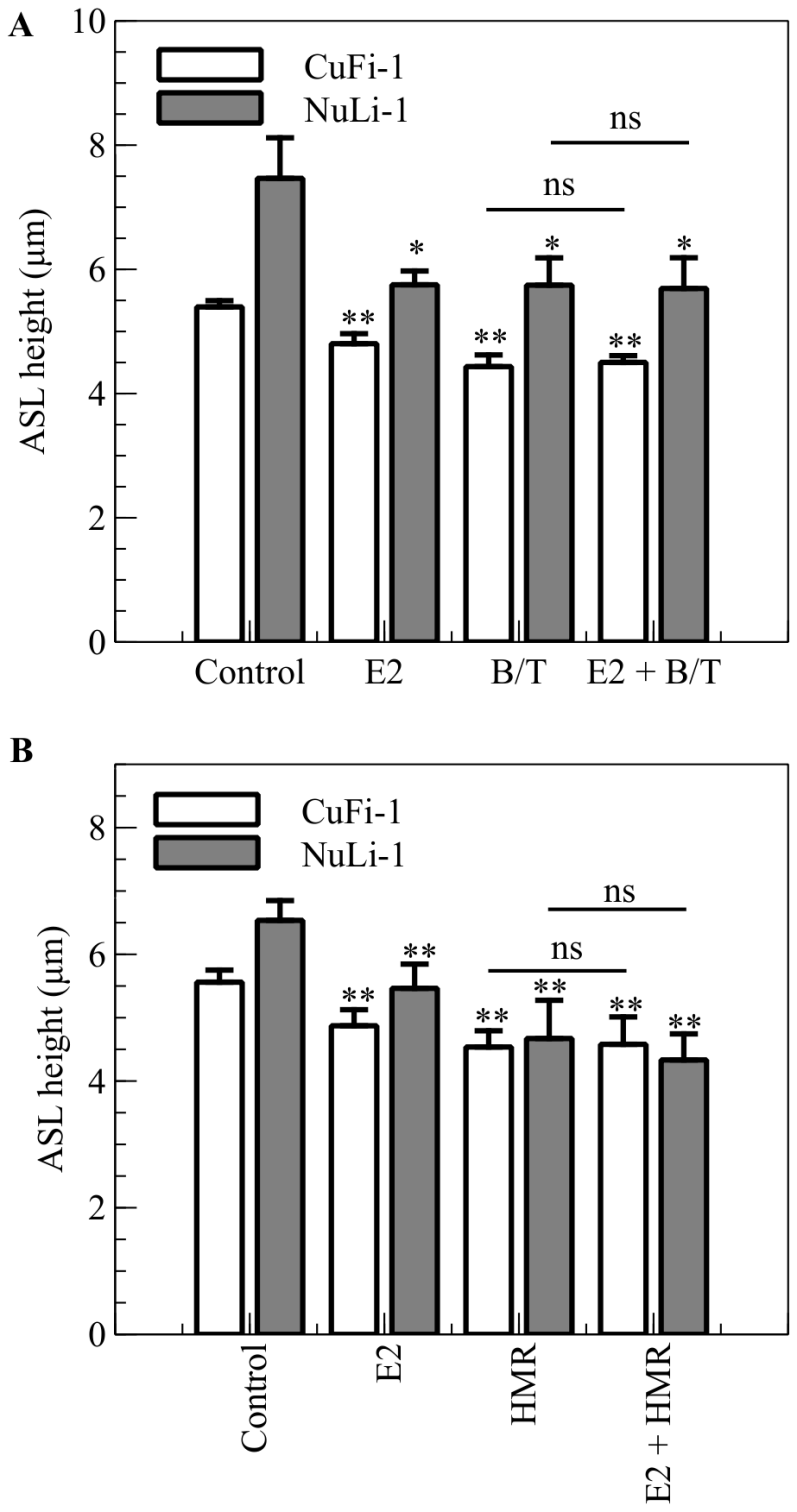
E2 inhibits K^+^ recycling in normal and CF bronchial cell lines. A. NuLi-1 (grey bars) and CuFi-1 (white bars) epithelial monolayers were stained with Calcein Green and ASL was stained with dextran conjugated to Texas Red^TM^ fluorochrome and ASL height was measured after treatment with E2 (30 min, 1 nM), and a cocktail of BaCl_2_ and TEA to inhibit potassium channels (B/T 30 min, BaCl_2_ 10 mM, TEA 1 mM) or pretreated with E2 and then treated with the K^+^ channels cocktail inhibitor (E2 + B/T) (n = 5, Error bars reflect standard error of the mean, ANOVA, * p<0.05, ** p<0.01). B. NuLi-1 (grey bars) and CuFi-1 (white bars) epithelial monolayers were treated with E2 (30 min, 1 nM), and the KCNQ1 channel specific inhibitor HMR1556 (HMR, 30 min, 1 µM) or pretreated with E2 and then treated with HMR1556 (E2 + HMR) (n≥3, Error bars reflect standard error of the mean, ANOVA, ** p<0.01).

It has been shown that K^+^ recycling at the basolateral membrane of epithelia regulates Cl^−^ secretion and Na^+^ absorption. The Na^+^/K^+^ ATPase transports K^+^ ions against an electrical and chemical gradient into the cell. Potassium is thus accumulated in the cell above its electrochemical equilibrium and is recycled across the basolateral cell membrane via K^+^ channels. The electrogenic leak of K^+^ maintains a membrane potential hyperpolarised in relation to the equilibrium potential for Cl^−^ and Na^+^, and thus maintains the electrical driving force for Cl^−^ secretion and Na^+^ absorption. Since we showed that E2 inhibited Cl^−^ secretion in NuLi-1 cells and activated Na^+^ absorption in CuFi-1 cells, the involvement of K^+^ channels in the regulation of ASL height and its response to estrogen were investigated. As a first approach, the total basolateral K^+^ conductance was reduced using a cocktail of K^+^ channel blockers containing BaCl_2_ (10 mM) and tetraethylammonium (TEA 1 mM). This combination of K^+^ channel blockers caused a significant decrease in ASL height in both NuLi-1 monolayers (from 7.5±0.7 µm to 5.8±0.4 µm, n = 5, p<0.05) and CuFi-1 monolayers (from 5.4±0.1 µm to 4.4±0.2 µm, n = 5, p<0.01). Subsequent estrogen addition had no additive effect on ASL height (E2 + BaCl_2_/TEA: 5.7±0.5 µm and 4.5±0.1 µm in NuLi-1 and CuFi-1, respectively), (Figure 5A). These results are highly suggestive that E2 also targets K^+^ recycling to decrease ASL height. We have previously reported that in distal colonic epithelium, Cl^−^ secretion is inhibited by E2 via the inhibition of the KCNQ1 potassium channel. Therefore, we examined if this channel was targeted by E2 in the airway epithelia by testing the effect of a KCNQ1 specific inhibitor, chromanol HMR1556, on ASL height. Chromanol HMR1556 (1 µM) significantly decreased ASL height in CuFi-1 monolayers (from 5.6±0.2 µm to 4.5±0.3 µm, n = 4, p<0.01) and in NuLi-1 monolayers (from 6.5±0.3 µm to 4.7±0.6 µm, n = 4, p<0.01), (Figure 5B). Subsequent treatment with estrogen had no additive effect, indicating that the KCNQ1 potassium channel is a target of E2 in bronchial epithelium as it is in colonic epithelium. Inhibition of KCNQ1 channels by estrogen will reduce the electrical driving force for Cl^−^ efflux across the apical membrane and may be an additional factor in mediating the E2 down-regulating effect on ASL height.

### Estrogen modulation of protein kinase activity in NuLi-1 and CuFi-1 cells

**Figure 6:**
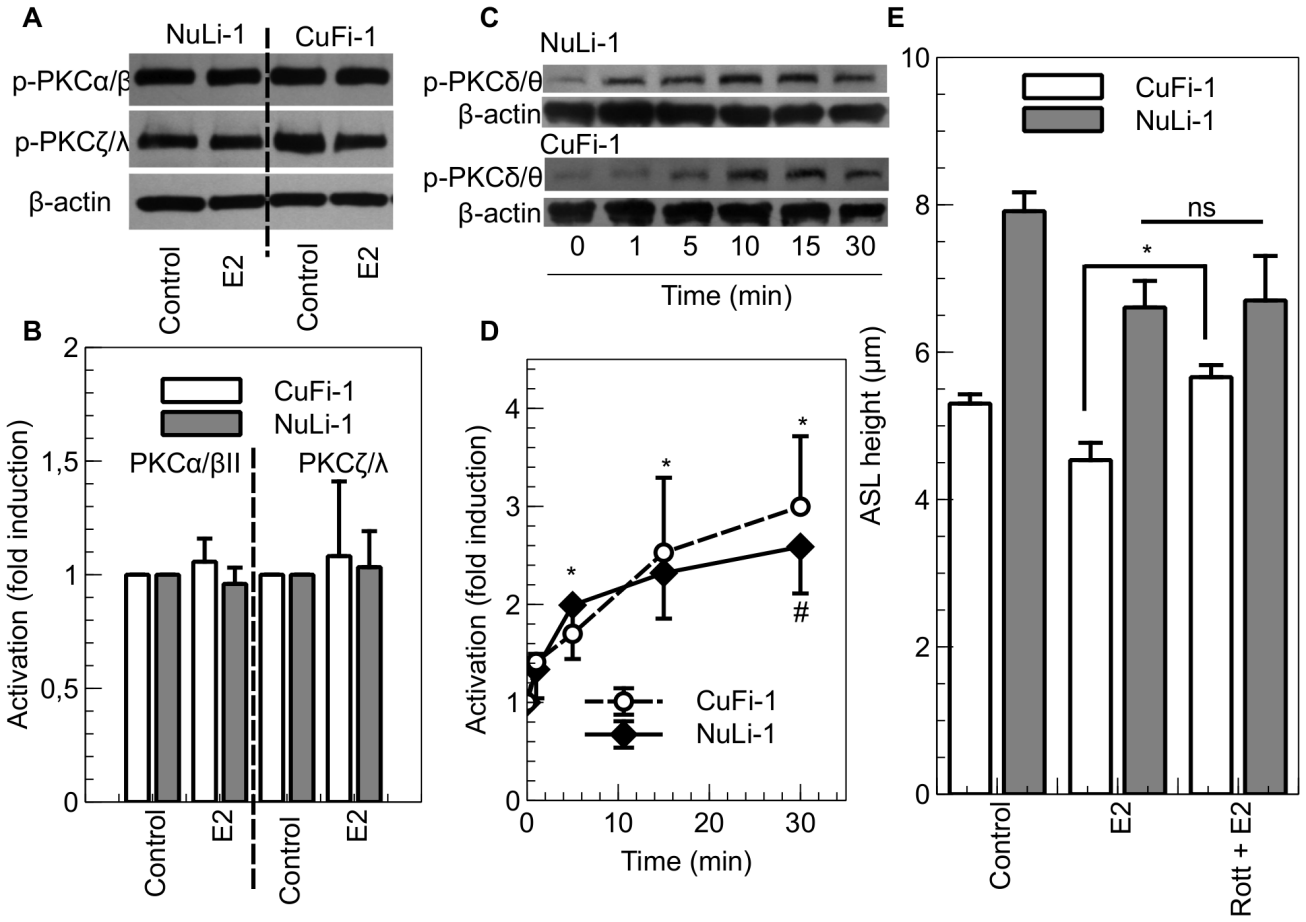
E2-induced PKCδ activation is responsible for the ASL height decrease in CuFi-1 cells. NuLi-1 (grey bars) and CuFi-1 (white bars) cells were treated basolaterally with E2 (1 nM) for 30 mins. After hormone treatment, cells were washed and total protein extracts were prepared in order to measure the level of phosphorylation of PKCα/βII and PKCζ/λ by immunoblot. Representative Western blots are shown in panel A and densitometric quantitation of phospho-PKCα/βII / β-actin and phospho-PKCζ/λ / β-actin are shown in panel B (n = 4, Error bars reflect standard error of the mean, Student’s t-test). B. NuLi-1 and CuFi-1 cells were treated basolaterally with E2 (1 nM) for the indicated times. After treatment, cells were washed and total protein extracts were prepared in order to measure the level of phosphorylation of PKCδ/θ. Representative images are shown in panel C and mean phosphorylation state of PKCδ/θ changes in control conditions, or following treatment with estrogen at different time points, are shown in panel D (n≥4, Error bars reflect standard error of the mean, ANOVA, * p<0.05). E. ASL height was measured in CuFi-1 (white bars) and NuLi-1 (grey bars) monolayers in response to estrogen and PKCδ inhibition. NuLi-1 and CuFi-1 monolayers were treated with E2 (1 nM) or pretreated with rottlerin (5 µM) and then treated with E2 (n = 5, Error bars reflect standard error of the mean, ANOVA, * p<0.05).

Our previous studies have shown the involvement of protein kinase C delta (PKCδ) activation in the regulation of intestinal ion transport by estrogen [13]. The effect of E2 on the activation of different PKC isoforms was assessed by Western blotting. As shown on Figure 6A and B, E2 had no effect on the phosphorylation of PKCα/βII and PKCζ/λ isoforms either in non-CF or in CF cells. However, E2 induced an increase in PKC δ/θ phosphorylation in NuLi-1 and CuFi-1 cells (Figure 6C) and this increase is significant in both cell lines after a 30 minutes treatment (Figure 6D). There was no effect of E2 treatment on the expression of these kinases (data not shown). To confirm the involvement of PKCδ/θ in the effect of E2 on ASL height, we tested a PKCδ specific inhibitor, rottlerin (5 µM), in ASL height measurement experiments. This inhibitor, alone, had no effect on ASL height in CF and non-CF cells (CF Control: 5.3±0.1 µm, rottlerin: 5.4±0.3 µm p>0.05; non-CF control: 7.9±0.23 µm, rottlerin: 8.2±0.7 µm, p>0.05, data not shown). However, a pre-treatment of CuFi-1 and NuLi-1 cells with rottlerin prior to the treatment with E2, prevented the E2-induced decrease in ASL height in CF CuFi-1 cells (from E2: 4.5±0.2 µm to rottlerin + E2: 5.7±0.2 µm, n  =  5 p< 0.05) without affecting the E2-induced decrease in ASL height in non-CF NuLi-1 cells (Figure 6E).

## Discussion

Female patients with CF have a worse prognosis than CF males, and many studies point towards a role for estrogen in the aetiology of this CF ‘gender gap’. It has been shown since the 1990s that estrogen exacerbates lung infection in CF. Estrogen increases the severity of *Pseudomonas aeruginosa*-induced pneumonia in CF mice [24] as well as promoting the conversion of this bacteria to a mucoid form in women with CF [22]. The airways in CF females are colonized earlier by pathogens such as *Burkholderia cepacia,* with a 2.4-increased risk compared to men with CF [35]. Moreover, in a study from 2010, Chotirmall *et al.* showed that estrogen inhibited the NF-κB signalling pathway, leading to a decrease in IL-8 secretion in CF bronchial epithelial cells. The combined effect of E2 on infection and inflammation was correlated with increased pulmonary exacerbations in female CF patients in high estrogen states (estrous mid-cycle) [21]. The CF gender gap tends to diminish with age, most likely related to decreased estrogen production as patients live longer and enter the menopause but also as a result of improved physiotherapy, more aggressive antibiotics and advanced care after lung transplant [36]. However, in spite of improved and aggressive therapies, females with CF still scored worse than age-matched CF males on quality of life studies [37], and they show more acute lung exacerbations [38]. It is therefore important to understand the basic cellular and molecular mechanisms by which estrogen can modulate lung function in women with CF so that adapted therapies can be implemented. In this study, we evaluated the role of estrogen on ASL dynamics and ion transport in airway epithelium. We found that physiological concentrations of estrogen decreased ASL height in both non-CF and CF bronchial epithelial cell lines but that it is still higher in non-CF cells than in CF cells. Estrogen decreased significantly the ASLh in primary epithelial cells grown from bronchial brushings obtained from CF children,. A previous study reported similar effects of E2 to reduce ASL in CF and non-CF airway cells via an inhibition of Ca^2+^ signalling [19]. Although the E2-induced decrease in ASLh might seem modest, the height of the ASL in normal human bronchial epithelium has been shown to be in the range of 7 to 9 µm and this height is critical for efficient mucociliary clearance. Indeed, the size of the outstretched cilia is approximately 7 µm and it has been shown that mucociliary clearance is impaired when ASL height is decreased [39]. In CF patients, the height of the airway surface liquid is already decreased and characterised by a reduced mucociliary clearance. During the menstrual cycle, E2 can reach concentrations of 1–1.5 nM at the pre-ovulation peak. Therefore, the 1 nM E2-induced decrease in ASL height from 5.6±0.1 µm to 4.4±0.3 µm in the CF cells (Figure 1B) would substantially further impair mucociliary clearance and could, at least in part, explain the increase in exacerbations observed in women with CF at this phase of the menstrual cycle [22]. Our study focused on the determination of the ion transporter targets, and we showed that E2 decreased ASL height in non-CF cells through the inhibition of cAMP mediated Cl^−^ secretion, indicating CFTR as the target of E2, which is consistent with previous studies showing the inhibition of this Cl^−^ channel after exposure to E2 [14]. It has also been shown that estrogen can regulate the expression of CFTR in the female reproductive tract [40] and affect its expression on developing fetal rat lung epithelium [41]. The effect of estrogen on CFTR is supported by the abolition of the effect of E2 in decreasing ASL in non-CF cells after increasing the intracellular levels of cAMP using forskolin (an activator of the enzyme adenylyl cyclase).

In CF cells, in which cAMP-activated Cl^−^ secretion is absent, the effect of estrogen to decrease the ASL height was mediated by enhanced sodium absorption. The evidence points to a rapid estrogen activation of ENaC as well as the Na^+^/K^+^ ATPase leading to a transepithelial hyperabsorption of Na^+^. Moreover, estrogen preincubation prevented the amiloride-induced increase in ASL height, confirming the involvement of ENaC in the effect of estrogen on ASL homeostasis. It was shown before that estrogen upregulates ENaCα and ENaCγ subunit mRNA and protein expression levels in osteoblasts, as well as increasing ENaC current in these cells [42]. In the lung, Laube *et al.* reported recently that estrogen increased Na^+^ transport in alveolar cells through the upregulation of the expression of ENaC and Na^+^/K^+^ATPase [20]. Taken together with our results, these data show that Na^+^ transport is an important target for estrogen regulation of fluid homeostasis in the lung. In our study, we also showed that estrogen exposure of CF and non-CF bronchial epithelial cells produced an increase in PKCδ/θ activation and that the specific inhibition of PKCδ prevents the effect of estrogen on ASL homeostasis in the CF cells. Although an increase in PKCδ/θ phosphorylation was observed in non-CF cells following treatment with E2, rottlerin did not prevent E2-induced ASLh decrease in these cells, showing that this kinase is either not involved in the modulation of ion transport by E2 in non-CF cells or that E2 activates alternative signalling pathways that could compensate for the effect of PKCδ activation on ASLh. The regulation of Na^+^ transport by PKC isozymes has been shown in different studies. For example, estrogen produced a 50% inhibition of ^22^Na^+^ uptake in pig kidney epithelial LLC-PK1 cells after PMA (PKC activator) treatment [43]. In the alveoli, it has been shown that PKC is involved in ENaC-mediated lung liquid regulation [44]. However, to our knowledge, no report has shown that the PKCδ isoform could regulate fluid absorption through the bronchial epithelia. Our data also demontrate an effect of E2 on the activity of Na^+^/K^+^ATPase, which may be transduced via PKCδ. In a recent study, Li *et al.* showed that estrogen regulates the expression of N-myc downstream-regulated gene 2 (NDRG2), a protein which can interact with the β1 subunit of the Na^+^/K^+^ATPase, stabilize it and thus increase the Na^+^/K^+^ ATPase-mediated Na^+^ transport [45]. Moreover, the PKCθ isoform, which displays the highest homology to PKCδ, is able to phosphorylate NDRG2 in C2C12 skeletal muscle cells [46]. The similarity between E2 effects on Cl^−^ secretion in distal colon [9] and here in normal bronchial epithelium points to a common signalling pathway (PKA) and ion channel target (KCNQ1). In CF cells, the signalling pathway involves PKCδ similar to female distal colon [13] and kidney cortical collecting duct cells (Robles, unpublished). In the latter study, E2 inhibited ENaC via PKCδ. We thus reason that the E2 anti-secretory pro-absorptive responses are a common feature in epithelial cells of lung, kidney and intestine [47].

Finally, we reported in this study that E2 acted on the ASL homeostasis very rapidly (within 30 minutes). The effect of E2 on ASL height was mimicked when both non-CF and CF cells were treated with the Estrogen Dendrimer Conjugate (EDC), whereas the empty dendrimer showed no effect on ASL fluid dynamics. These results point to a rapid and non-genomic effect of 17β-estradiol in these bronchial epithelial cells. In the classic genomic pathway, E2 binds to one of its receptors (ERα or ERβ), which dimerizes and interacts at chromatin gene-regulatory sites, where this complex recruits co-factors and modulates the transcription of target genes. These direct transcriptional effects can be measured after a few hours. The non-genomic pathway, on the other hand, can involve both types of receptors and in particular a membrane-associated palmitoylated form of ER [48], [49], which induces the up or down regulation of the activity of cytosolic protein kinases, such as PKA, PKC or the MAPKinases. This process is rapid (initiated within seconds) and can regulate ion transport by activating or inhibiting ion channels. Both isoforms of estrogen receptors (α and β) can be localized at the plasma membrane and form dimers [50], [51]. Although it is unclear through which extranuclear receptor, E2 and the EDC might exert their effect on ASLh, there is growing evidence for the involvement of ERα mediated actions of the EDC [26], [52], [53], [54]. In CuFi and NuLi cell lines, the predominant form of ERα is the 36kDa isoform (ER-α36) as shown on the Western blot (Methods S1- Figure S2A). It has been shown that ER-α36 mainly localises in the cytoplasm and at the plasma membrane [55], [56]; This ER isoform has been shown to mediate E2-stimulted PKCδ activation in endometrial Ishikawa cancer cells [57]. Although the localization of this receptor at the membrane could not be clearly established by immunofluorescence (Methods S1) using the same antibody as for the immunoblots (Methods S1), in CuFi-1 and NuLi-1 cells, ERα is clearly located to the cytoplasm and treatment with E2 did not induce its translocation to the nucleus over the 30 minute observation period (Figure S2B). ERβ was detected in both cell lines (Figure 2C) although its molecular weight (45 kDa) appeared lower than predicted (59 kDa). There was no difference in the localization of ERβ between CF and non-CF cells and short-term treatment with estrogen did not affect ERβ localization (Figure S2D). Although our study demonstrates a rapid effect of E2 (and EDC) on the ASL fluid dynamics, one should not conclude that no genomic effect is induced during this process. Indeed, it has been shown ‘non-genomic’ E2 responses both can prime and sustain transcriptional events through non-nuclear ER responses. Thus, non-genomic effects of EDC can generate genomic responses [34] transduced via activation of the transcription factor CREB [54].

Taken together with these studies, our results strongly suggest that the effect of E2 on ASL fluid dynamics and ion transport is mediated through an extranuclear ERα. The further investigation of the involvement of each isoform of the estrogen receptor as well as the long term effect of E2 and EDC on ASLh and gene transcription will be beneficial in order to fully understand the deleterious effect of estrogen in women with CF.

In conclusion, we have identified the ion channel targets for estrogen in non-CF and CF bronchial epithelia and the consequences for maintaining airway surface liquid homeostasis. Moreover, a non-genomic rapid response to estrogen transduced via extranuclear ER is a component of the deleterious effects of 17β-estradiol in CF lung pathophysiology.

## Supporting Information

Figure S1 **Contribution of Na^+^ absorption and Cl** ^−^ **secretion to total Isc in NuLi-1 and CuFi-1 cells.** Amiloride and bumetanide-sensitive currents were measured in NuLi-1 and CuFi-1 cell monolayers mounted in Ussing chambers. Panel A shows amiloride and bumetanide-sensitive currents inµAmp.cm^−2^ (n≥8, error bars reflect standard error of the mean, Student’s t-test, *** p<0.001). Panel B shows the ratio between amiloride-sensitive current and the total current as a percentage (n≥17, error bars reflect standard error of the mean, Student’s t-test, *** p<0.001) and the ratio between bumetanide-sensitive current and the forskolin-induced current as a percentage (n≥8, error bars reflect standard error of the mean, Student’s t-test, *** p<0.001).
(TIF)

Figure S2 **Expression pattern and localization of ERα and ERβ isoforms in NuLi-1 and CuFi-1 cells.** Panel A shows the expression of the different isoforms of ERα in NuLi-1 and CuFi-1 cells by Western blot. Panel B shows representative images of the intracellular localization of ERα before and after 30 mins treatment with 1 nM E2 (n = 4, blue, DAPI; green, ERα; red, actin). Panel C shows the expression of ERβ in NuLi-1 and CuFi-1 cells by Western blot. Panel D shows representative images of the intracellular localization of ERβ before and after 30 mins treatment with 1 nM E2 (n = 4, blue, DAPI; green, ERβ; red, actin).
(TIF)

Methods S1 **The detailed methods for Estrogen Receptors Immunoblotting and Immunofluoresence detection are detailed in Methods S1.**
(DOC)
