# Supplementary figures and images for: Non-Genomic Estrogen Regulation of Ion Transport and Airway Surface Liquid Dynamics in Cystic Fibrosis Bronchial Epithelium
Source: PLoS One. 2013 Nov 4;8(11):e78593. doi: 10.1371/journal.pone.0078593 (PMC3817220; doi:10.1371/journal.pone.0078593)

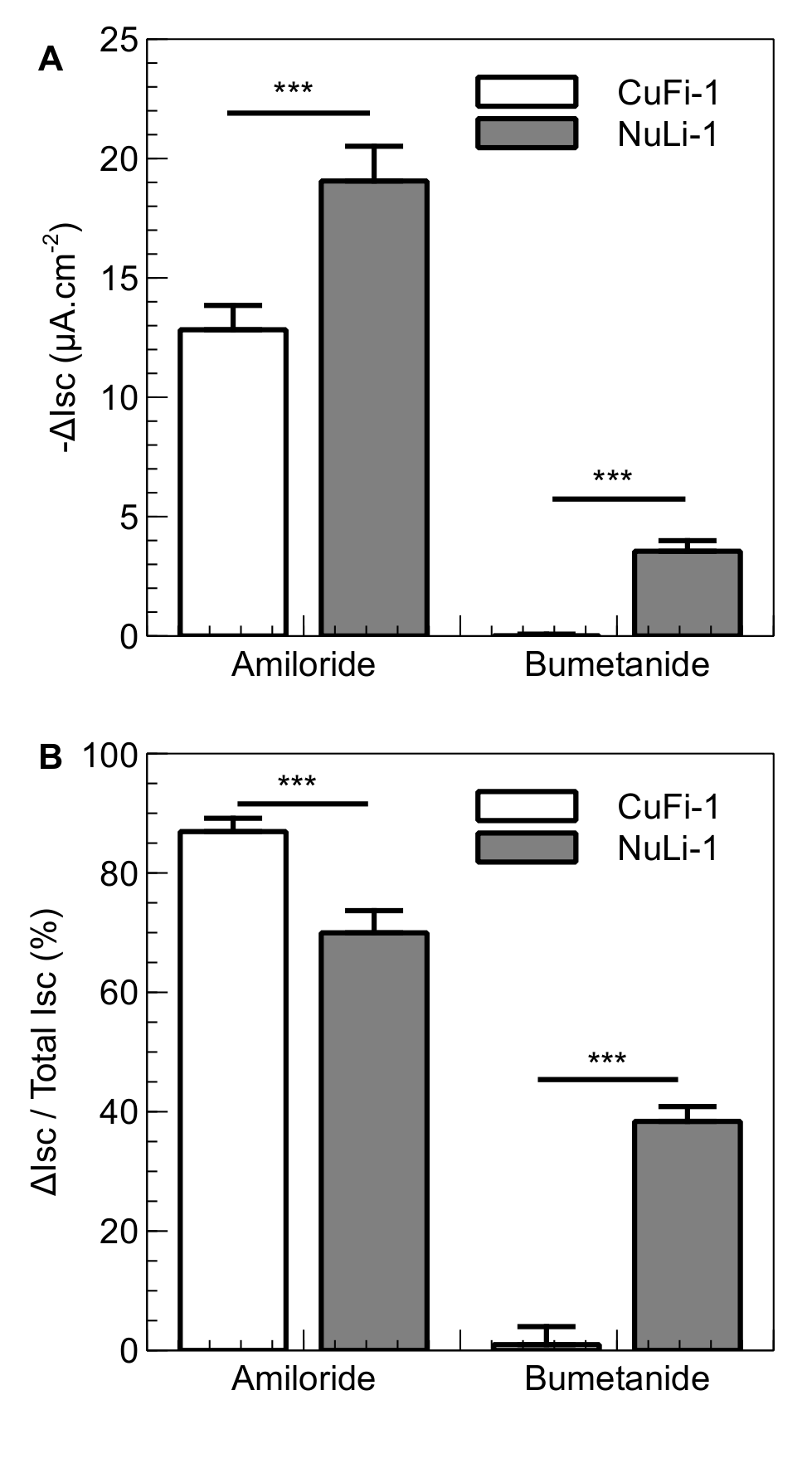

Supplement: Figure S1 — Contribution of Na+ absorption and Cl − secretion to total Isc in NuLi-1 and CuFi-1 cells. Amiloride and bumetanide-sensitive currents were measured in NuLi-1 and CuFi-1 cell monolayers mounted in Ussing chambers. Panel A shows amiloride and bumetanide-sensitive currents inµAmp.cm−2 (n≥8, error bars reflect standard error of the mean, Student’s t-test, *** p<0.001). Panel B shows the ratio between amiloride-sensitive current and the total current as a percentage (n≥17, error bars reflect standard error of the mean, Student’s t-test, *** p<0.001) and the ratio between bumetanide-sensitive current and the forskolin-induced current as a percentage (n≥8, error bars reflect standard error of the mean, Student’s t-test, *** p<0.001). (TIF) [file pone.0078593.s001.tif]

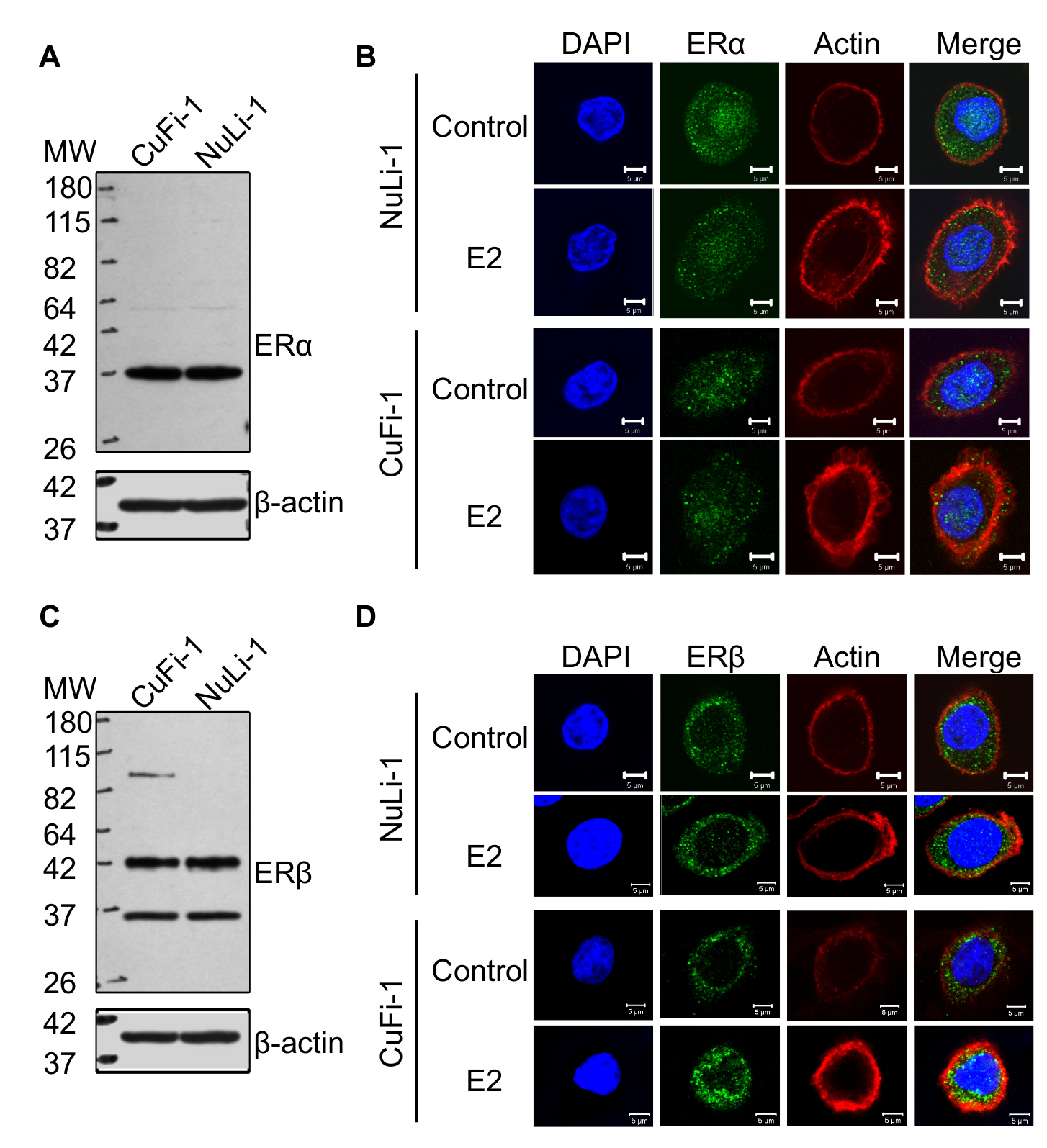

Supplement: Figure S2 — Expression pattern and localization of ERα and ERβ isoforms in NuLi-1 and CuFi-1 cells. Panel A shows the expression of the different isoforms of ERα in NuLi-1 and CuFi-1 cells by Western blot. Panel B shows representative images of the intracellular localization of ERα before and after 30 mins treatment with 1 nM E2 (n = 4, blue, DAPI; green, ERα; red, actin). Panel C shows the expression of ERβ in NuLi-1 and CuFi-1 cells by Western blot. Panel D shows representative images of the intracellular localization of ERβ before and after 30 mins treatment with 1 nM E2 (n = 4, blue, DAPI; green, ERβ; red, actin). (TIF) [file pone.0078593.s002.tif]
