# Supplementary material for: Non-Genomic Estrogen Regulation of Ion Transport and Airway Surface Liquid Dynamics in Cystic Fibrosis Bronchial Epithelium
Source: PLoS One. 2013 Nov 4;8(11):e78593. doi: 10.1371/journal.pone.0078593 (PMC3817220; doi:10.1371/journal.pone.0078593)
Supplement: Methods S1 — The detailed methods for Estrogen Receptors Immunoblotting and Immunofluoresence detection are detailed in Methods S1. (DOC) [file pone.0078593.s003.doc]

**Methods S1**

**Immunoblotting**

Total cell protein from untreated NuLi-1 and CuFi-1 cells were extracted and immunoblotting performed as described in Materials and Methods, using the following antibodies: anti-ESR1 (ERα, ARP31088, dilution 1/1000, Aviva Systems Biology Corp. San Diego, CA) anti-ESR2 (ERβ, ARP32258, dilution 1/1000, Aviva Systems Biology Corp. San Diego, CA).

**Immunofluorescence**

NuLi-1 and CuFi-1 cells grown on glass coverslips were incubated for 30 mins with or without E2 (1 nM), washed twice with PBS, and fixed in 4 % paraformaldehyde in PBS for 20 mins at room temperature. Fixed cells were then washed 3 times in PBS, permeabilized in 0.1% Triton X-100 in PBS for 15 mins at room temperature, rinsed 3 times in PBS and blocked in 10% goat serum in PBS for 1 h at room temperature. Cells were then incubated overnight at 4°C with the primary antibody (ESR1 or ESR2, dilution 1/200). Coverslips were then rinsed in 1% goat serum in PBS 3 times for 10 mins, incubated for 2 h at room temperature with the secondary antibody (goat anti-rabbit AlexaFluor 488 1/2000) and rhodamine-phalloïdin (dilution 1/3000) before being rinsed in 1% block solution for 3x10 mins, away from light, and mounted using DAPI-containing Vectashield. Coverslips were allowed to dry overnight at 4°C before the images were recorded on a Zeiss confocal Laser Scanning Microscope 710.
